# Quaternary ice sheets and sea level regression drove divergence in a marine gastropod along Eastern and Western coasts of South America

**DOI:** 10.1038/s41598-020-57543-4

**Published:** 2020-01-21

**Authors:** P. J. Fernández Iriarte, C. A. González-Wevar, N. I. Segovia, S. Rosenfeld, M. Hüne, L. Fainburg, J. D. Nuñez, P. A. Haye, E. Poulin

**Affiliations:** 1 IIMyC, Instituto de Investigaciones Marinas y Costeras, CONICET – FCEyN, Universidad Nacional de Mar del Plata, Funes 3250 (7600), Mar del Plata, Provincia de Buenos Aires, Argentina; 2 Laboratorio de Genómica y Ecología Molecular Antártica y sub-Antártica, Instituto de Ciencias Marinas y Limnológicas (ICML), Universidad Austral de Chile, Casilla # 567, Valdivia, Chile; 3 Centro FONDAP de Investigación en Dinámica de Ecosistemas Marinos de Altas Latitudes (IDEAL), Universidad Austral de Chile, Casilla # 567, Valdivia, Chile; 4 Instituto de Ecología y Biodiversidad (IEB), Departamento de Ciencias Ecológicas, Facultad de Ciencias, Universidad de Chile, Las Palmeras #, 3425 Ñuñoa, Santiago Chile; 5 Laboratorio de Diversidad Molecular, Departamento de Biología Marina, Facultad de Ciencias del Mar, Universidad Católica del Norte, Coquimbo, Chile; 6 Laboratorio de Ecosistemas Marinos Antárticos y Subantárticos, Universidad de Magallanes, Bulnes, 01890 Punta Arenas, Chile

**Keywords:** Population genetics, Evolutionary biology

## Abstract

The southern coastline of South America is a remarkable area to evaluate how Quaternary glacial processes impacted the demography of the near-shore marine biota. Here we present new phylogeographic analyses in the pulmonate *Siphonaria lessonii* across its distribution, from northern Chile in the Pacific to Uruguay in the Atlantic. Contrary to our expectations, populations from the southwestern Atlantic, an area that was less impacted by ice during glacial maxima, showed low genetic diversity and evidence of recent expansion, similar to the patterns recorded in this study across heavily ice-impacted areas in the Pacific Magellan margin. We propose that Atlantic and Pacific shallow marine hard-substrate benthic species were both affected during the Quaternary in South America, but by different processes. At higher latitudes of the southeast Pacific, ice-scouring drastically affected *S. lessonii* populations compared to non-glaciated areas along the Chile-Peru province where the species was resilient. In the southwest Atlantic, *S. lessonii* populations would have been dramatically impacted by the reduction of near-shore rocky habitat availability as a consequence of glacio-eustatic movements. The increase of gravelly and rocky shore substrates in the southwest Atlantic supports a hypothesis of glacial refugia from where the species recolonized lower latitudes across the Atlantic and Pacific margins. Our results suggest that current patterns of genetic diversity and structure in near-shore marine benthic species do not solely depend on the impact of Quaternary glacial ice expansions but also on the availability of suitable habitats and life-history traits, including developmental mode, bathymetry and the likelihood of dispersal by rafting.

## Introduction

Quaternary ice sheet expansion and contraction during the Last Glacial Maximum (LGM), between 23,000 and 18,000 years before present, triggered major climate and environmental changes that strongly affected the distribution of the biota worldwide^1,2^. Under the Expansion-Contraction (E-C) model of Pleistocene biogeography, cold-temperate species contracted their distribution ranges towards lower latitude glacial refugia located in less ice-impacted or non-glaciated areas^3^. During interglacial periods they recolonized higher latitudes through range expansions following deglaciation processes^4–6^. This simple E-C model provides a straightforward paradigm to test population demographic hypotheses through the Quaternary, and phylogeographic studies have helped to understand better the response of species to major climate changes during this period by recognizing distribution range shifts, potential refugial areas and recolonization routes^7–9^.

In southern South America, glacial periods produced major shifts in sea level, climate and landscapes, particularly in marine near-shore environments^10^. During the LGM, the Pacific margin of the Magellan province, from Chiloé Island (42°S) to Cape Horn (56°S), was almost completely covered by the Patagonian Ice Sheet^10–12^. Consequently, the availability of near-shore sheltered and hard-substrate habitats in the Pacific Magellan margin became very limited or even absent, and accordingly impeded the persistence of numerous near-shore marine species. Heavily ice-impacted areas were recolonized as the ice receded by dispersal of individuals from refugial populations. Repetitive rounds of local extinctions followed by recolonization may have enhanced the diversification of invertebrates across the region by the availability/diversity of vacant habitats and/or by survival in multiple refugia that may have led to allopatric divergence^13,14^.

In contrast to the glacial history recorded along the Pacific Magellan margin, ice sheet expansion and contraction would have affected the marine ecosystems across the southwestern Atlantic to a much lesser extent^15^. Accordingly, near-shore marine species in the Atlantic Magellan margin could have persisted *in situ* during the last glacial period, in contrast to their conspecific populations located in the Pacific.

During the last two decades phylogeographic and population-based studies have been carried out for different groups of Magellan freshwater^16,17^ and near-shore marine^18–24^ organisms. Phylogeographic studies in freshwater and terrestrial Magellan ecosystems have inferred the presence of Quaternary glacial refugia on the east side of the Andes^25^, as well as on the Pacific Magellan margin within^17,26^ and outside the glacier limits^19,27^. Phylogeographic studies in Magellan marine organisms have established that most of the taxa analyzed exhibited low levels of genetic diversity, absence of genetic structure and strong signals of recent demographic growth, supporting the hypothesis of postglacial expansions^19–23,27,28^. Nevertheless, although several near-shore marine species are broadly distributed across the whole Magellan province, very few phylogeographic studies have been conducted across the whole region^21,22,29^. In fact, most of the studies have been restricted either to the Pacific^18,19,29,30^ or to the Atlantic^23,31^ margin of the Magellan province. Therefore, a phylogeographic study conducted on a broadly distributed species would represent an important contribution to propose an integrated scenario of how coastal marine biota have responded to historical climate changes along temperate and cold areas of southern South America.

Members of the pulmonate genus *Siphonaria* are distributed worldwide, except in the Arctic. In the last revision of the genus, Dayrat *et al*.^32^ recognized three distinct species in southern South America: *Siphonaria lessonii* Blainville, 1827, *S. lateralis* Gould, 1846, and the recently described *S. fuegiensis*^33^. *Siphonaria lessonii* is commonly found on intertidal rocky ecosystems and is widely distributed from southern Peru to Cape Horn in the Pacific and north to Uruguay in the Atlantic, as well as in the Malvinas/Falkland Islands^34^. Accordingly, the distribution of this pulmonate limpet extends across different ecoregions^35^ in temperate areas of the Chile-Peru and the Argentina provinces, as well as cold areas across the Magellan Province^36^. The species *S. lessonii* is a hermaphroditic broadcast-spawner with free-swimming veliger larvae that disperse for approximately 8 to 11 days in the water column^37^. Considering the geographic range and the reproductive biology of the species, *S. lessonii* is a suitable model to infer the impact of Quaternary glacial cycles over its distribution range, demographic inferences, recolonization pathways and its local persistence across a latitudinal gradient in areas that were differentially affected by the Quaternary glacial processes.

A first phylogeographic genetic approach based on a fragment of the mitochondrial DNA (mtDNA) COI gene recognized the presence of two genetic clades in the species, one on the Pacific margin of South America and one on the Atlantic side, which would have diverged between 100,000 and 1,000,000 years ago^24^. However, the low number of samples from the Pacific margin did not allow them to locate the actual limit between the recorded genetic clades. More recently, new phylogeographic analyses in the species were combined with environmental niche modelling analyses along the southeast Pacific coast^28^. The results of this study are congruent with a historical scenario involving Quaternary demographic and distribution contractions of *S. lessonii* surviving in glacial refugia in the southern portion of the southeastern Pacific, followed by recolonization of the deglaciated areas. Nevertheless, the absence of information concerning the genetic diversity of the species along its Atlantic range may result in an incomplete or misinterpreted Quaternary demographic scenario. Therefore, a broader phylogeographic study of *S. lessonii* is required to better understand the historical biogeography of a species widely distributed across the southern Atlantic and Pacific coasts of South America.

This study aims to evaluate the impacts of past glaciations on marine taxa using as a model species the pulmonate *Siphonaria lessonii* across different South American provinces. For this purpose we performed phylogeographic and population-based analyses using fragments of the mitochondrial cytochrome oxidase c subunit 1 (COI) and the nuclear internal transcribed spacers (ITS1 and ITS2). We hypothesize that Quaternary glacial processes have differentially affected *S. lessonii*, since habitat eradication would have strongly impacted populations along Pacific coasts. In contrast, southwestern Atlantic populations may have persisted through glacial cycles, as previously demonstrated in the Chile-Peru Province. Accordingly, populations from the Magallanes Province, and particularly those from the Pacific margin are expected to show lower levels of genetic diversity than temperate populations from the Chile-Peru and Argentina provinces, and should harbor a genetic signal of a recent demographic expansion process. Conversely, lower latitude populations located in non-glaciated areas of the southwestern Atlantic and the Chile-Peru province are expected to exhibit higher levels of genetic diversity and structure as a consequence of more stable demographic histories. Hence, we expect to find different genetic signatures between higher latitude Magellan populations and lower latitude temperate populations located across the Atlantic and Pacific South American margins.

## Results

### Sample collection and genetic polymorphism in *S. lessonii*

**Figure 1:**
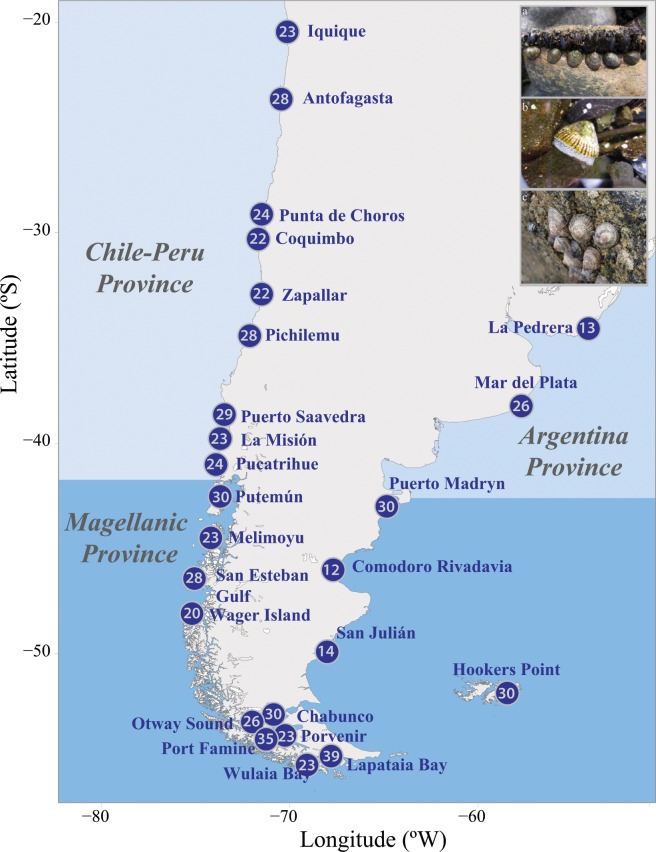
Sampling localities of *Siphonaria lessonii* across its distribution in different provinces of the southeastern Pacific and the southwestern Atlantic. Shapefiles of the coastlines available in the database of GEOdas (NOAA) were filtered using GEOdas Coastline Extractor v. 1.1.3.1. (https://www.ngdc.noaa.gov/mgg/geodas/geodas.html). Photographs of intertidal *S. lessonii* courtesy of co-authors Pilar Haye (a & b) and Sebastian Rosenfeld (c).

*Siphonaria lessonii* individuals were collected from 25 localities across the species’ distribution in three main South American biogeographic areas as defined by Trovant *et al*.^29^ (Fig. 1).

The complete COI data set of *S. lessonii* included 625 individuals and consisted of 565 nucleotide positions coding for 188 amino acids. Out of the analyzed COI sequences, 563 are new for the species and the rest (n = 62) were included in the study of Nuñez *et al*.^25^ No insertion, deletion, or stop codons were detected in the whole COI data set. The complete ITS data set included 45 new sequences, consisted of 1259 nucleotides and a total of six insertion/deletions were recorded. Mitochondrial and nuclear sequences were not saturated at any position and within the COI data set we recorded four amino acid substitutions (positions 6, 31, 100, and 117). High levels of mtDNA genetic variability characterized *S. lessonii* with 115 polymorphic positions (20.35%), of which 66 (57.39%) were parsimoniously informative. Mitochondrial DNA sequences were A-T rich (63.5%) compared to the mean G-C content. Conversely, nuclear ITS sequences were G-C rich (59.3%) and more conserved than mtDNA sequences, with only 24 polymorphic positions (1.90%) of which 13 (54.1%) were parsimoniously informative.

### Main patterns of genetic structure in *S. lessonii* across its distribution

**Figure 2:**
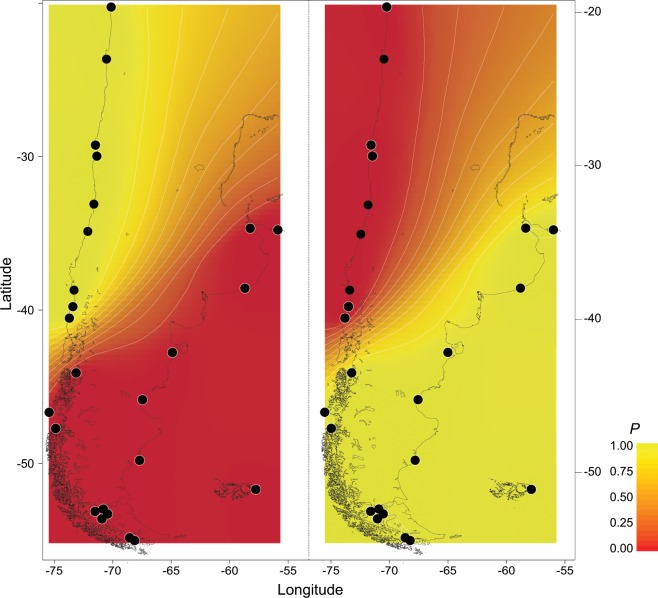
Spatial genetic structure of *Siphonaria lessonii* across its distribution on the southeastern Pacific and the southwestern Atlantic based on GENELAND analyses. The black circles indicate the relative positions of the sampling localities. Dark and light shades are proportional to posterior probabilities of membership in clusters. Yellow and red areas show the highest and lowest probabilities of membership in clusters, respectively. Posterior probabilities were plotted against the southern South America coastline shapefile plotted in the database of GEOdas (NOAA) and filtered using GEOdas Coastline Extractor v 1.1.3.1 (https://www.ngdc.noaa.gov/mgg/geodas/geodas.html).

SAMOVA and GENELAND analyses recovered similar patterns of phylogeographic structure in *S. lessonii*, with the presence of two main genetic groups. SAMOVA recovered two significantly differentiated groups (F_CT_ = 0.64) explaining 64.48% of the total variance. In contrast, differences between localities within groups were significant (F_SC_ = 0.03) but only explained 1.35% of the total variation (Table 1). The first genetic group (C1) included populations along the Chile-Peru Province (CP) north of 42°S, between Iquique (20°S) and Pucatrihue (40°S). The second genetic group (C2) included sites located south of 42°S along the Magellan province together with localities from the Argentina province, as well as the Malvinas/Falkland Islands (MFI). The Bayesian clustering algorithm detected the same phylogeographic structure with the recognition of two main groups (K = 2) (Fig. 2A,B). Values for group membership were very high for all localities (c.a. P = 0.95). The mean probability value (P = 0.5), which corresponds to the boundary between these genetic groups, crossed the Pacific coast at approximately 42°S (Fig. 2). Again, these two genetic groups included 1) CP localities and 2) MP + MFI and AP localities (Fig. 2). The nearest neighbor statistic algorithm in the complete *S. lessonii* COI data set (S_nn_ = 0.11) detected a low but significant phylogeographic signal (P < 0.001). However, when this analysis was performed considering the SAMOVA and GENELAND groupings (C1 *vs*. C2), S_nn_ became very high (S_nn_ = 0.87) and remained highly significant (P < 0.0001), demonstrating the high phylogeographic signal detected in the species across its distribution.

**Table 1:** Spatial Analysis of Molecular Variance (SAMOVA) across *Siphonaria lessonii*’s distribution depicting the percentage of variation explained among groups (C1 and C2) among populations within groups, and within populations.

Source of variation	d.f.	Sum of squares	Variance components	Percentage of variation
Among groups	1	810.655	2.81539 Va	64.48
Among populations within groups	23	68.000	0.05881 Vb	1.35
Within populations	600	895.460	1.49243 Vc	34.18
Total	624	1774.115	4.36663	

F_SC_ = differentiation within populations among groups and F_CT_ = differentiation among groups (**p < 0.01, ***p < 0.001). R1 = Chile-Peru Province; R2 = Magellan Province + Argentina Province.

Fixation Index

F_SC_: 0.03791***

F_CT_: 0.64475***.

### Contrasting patterns of genetic diversity structure in *S. lessonii* across its distribution

Levels of mtDNA genetic diversity were significantly higher in R1 populations across the Chile-Peru province (CP) compared to those recorded south of 42°S, across MP and AP within R2 (Table 2). Extremely low levels of genetic diversity recorded at La Pedrera, Uruguay, Comodoro Rivadavia, and San Julián, Argentina, must be taken with precaution considering the reduced number of individuals analyzed in these sites (12, 14, and 13 respectively, Table 2). Accordingly, these localities were excluded from subsequent comparative analyses. Haplotype diversity (*H*) varied between 1.000 (Iquique, CP) and 0.418 (Hookers Point, MFI). The number of polymorphic sites (*S*) varied between 27 (Iquique, CP) and 7 (Wulaia Bay, MP). Similarly, the number of haplotypes (*k*) varied between 30 (Iquique, CP) and 6 (Wager Island and Porvenir, MP) (Table 2). The average number of nucleotide differences (*Π*), and the mean nucleotide diversity (*π*) varied between 6.53/0.0115 (La Misión, CP) and 0.6/0.001 (Wulaia, MP) (Table 2).

**Table 2:** Geographical coordinates, diversity indices and neutrality tests (Tajima’s D and Fu’s F_S_) in *Siphonaria lessonii* populations across its distribution in the southeast Pacific and the southwest Atlantic.

Locality	Lat.	Long.	*N*	*k*	*H*	*S*	*Π*	*π*	D	F_S_
Iquique	−20.25	−70.13	23	23	1.000	27	4.22	0.007	−1.82*	−26.76***
Antofagasta	−23.48	−70.40	28	21	0.976	26	3.52	0.006	−1.70	−16.76***
Punta de Choros	−29.25	−71.46	24	17	0.938	34	4.38	0.007	−1.97*	−8.75***
Coquimbo	−29.96	−71.33	22	14	0.948	24	3.81	0.006	−1.58	−5.92**
Zapallar	−32.55	−72.45	22	15	0.952	22	3.23	0.005	−1.73	−8.73***
Pichilemu	−34.41	−72.03	28	22	0.976	31	5.39	0.009	−1.18	−13.69***
Puerto Saavedra	−38.78	−73.33	29	23	0.975	37	5.70	0.010	−1.57	−14.39***
La Misión	−39.78	−73.40	23	18	0.972	29	6.53	0.011	−0.64	−7.76***
Pucatrihue	−40.53	−73.70	24	17	0.960	23	5.56	0.009	−0.35	6.82**
Cluster 01			223	111	0.977	84	4.99	0.008	−2.00*	−158.818***
Putemún	−42.41	−73.75	30	13	0.648	20	3.10	0.005	−1.34	−4-04*
Melimoyu	−44.10	−75.43	23	13	0.818	22	5.06	0.008	−0.59	−2.69*
San Esteban Gulf	−46.66	−74.86	28	19	0.881	28	5.52	0.009	−0.84	−8.04***
Wager Island	−47.71	−74.87	20	6	0.516	15	3.18	0.005	−0.91	1.08
Port Famine	−53.61	−70.92	35	11	0.496	20	1.62	0.002	−2-25**	−4.89**
Otway Sound	−53.13	−71.50	26	10	0.748	11	1.20	0.002	−1.93*	−6.33**
Chabunco	−52.98	−70.80	30	10	0.517	10	0.72	0.001	−2.25**	−8.98***
Porvenir	−53.30	−70.43	23	6	0.395	19	2.04	0.003	−2.21**	0.02
Wulaia Bay	−55.03	−68.13	23	7	0.462	7	0.60	0.001	−2.14**	−5.25**
Lapataia Bay	−54.85	−68.55	39	13	0.560	14	1.00	0.001	−2.24**	−10.65***
San Julián	−49.80	−67.70	14	5	0.505	11	1.81	0.003	−1.91*	0.020
Comodoro Rivadavia	−45.85	−67.45	12	2	0.167	1	0.16	0.000	−1.14	−0.47
Puerto Madryn	−42.76	−64.90	30	9	0.632	19	2.807	0.004	−1.43	−0.91
Mar del Plata	−38.03	−57.51	26	9	0.578	8	0.757	0.001	−2.18**	−7.35***
La Pedrera	−34.58	54.11	13	1	0.000	0	0	0	n/a	n/a
Hookers Point	−51.70	−57.76	30	8	0.418	19	1.26	0.002	−2.55***	−2.97*
Cluster 02			402	93	0.568	78	2.13	0.003	−2.43***	−153.32***
Total			625	151	0.749	97	5.04	0.0091	−1.94*	−213.80***

Where: n = number of analyzed individuals; k = number of haplotypes; S = polymorphic sites; H = haplotype diversity; Π = average number of pairwise differences; π = nucleotide diversity.

*p < 0.05, **p < 0.01, ***p < 0.001.

Considering the high levels of genetic diversity recorded in R1 populations and the high number of single-frequency haplotypes recorded in this genetic group, the analyses of structure were performed using mean pairwise differences (N_ST_). Mean general values of differentiation were high, especially considering the mean N_ST_ = 0.314 (Table 3). Nevertheless, most of the differences were found comparing R1 *vs*. R2 localities. Sites across the Chile-Peru province (R1) showed low levels of genetic differentiation from Iquique (20°S) to Pucatrihue (40°S) (Table 3). Similarly, comparisons between R2 populations failed to recognize any significant structure in *S. lessonii* south of 42°S from Chiloé in the Pacific to Uruguay in the Atlantic (Table 3).

**Table 3:** Pairwise N_ST_ values calculated between the analyzed populations of *Siphonaria lessonii* (20,000 iterations). Statistical significant differences are marked in bold. Where: 1) Iquique; 2) Antofagasta; 3) Punta de Choros; 4) Coquimbo; 5) Zapallar; 6) Pichilemu; 7) Puerto Saavedra; 8) La Misión; 9) Pucatrihue; 10) Putemún; 11) Melimoyu; 12) San Esteban Gulf; 13) Wager Island; 14) Port Famine; 15) Otway Sound; 16) Chabunco; 17) Porvenir; 18) Wulaia; 19) Lapataia; 20) San Julián; 21) Comodoro Rivadavia; 22) Puerto Madryn; 22) Mar del Plata; 23) La Pedrera,; 25) Hookers Point.

	1	2	3	4	5	6	7	8	9	10	11	12	13	14	15	16	17	18	19	20	21	22	23	24	25
1	***																								
2	0.00	***																							
3	0.00	0.00	***																						
4	0.00	0.00	0.00	***																					
5	0.00	0.00	0.00	0.00	***																				
6	0.02	0.01	0.00	0.00	0.00	***																			
7	0.01	0.02	0.00	0.00	0.01	0.00	***																		
8	0.10	0.10	0.06	0.00	0.05	0.01	0.01	***																	
9	0.07	0.08	0.05	0.06	0.03	0.00	0.00	0.00	***																
10	**0.62**	**0.64**	**0.60**	**0.60**	**0.62**	**0.49**	**0.51**	**0.38**	**0.42**	***															
11	**0.45**	**0.48**	**0.42**	**0.42**	**0.44**	**0.31**	**0.33**	**0.20**	**0.23**	0.02	***														
12	**0.45**	**0.48**	**0.43**	**0.42**	**0.44**	**0.33**	**0.34**	**0.22**	**0.24**	0.02	0.00	***													
13	**0.60**	**0.63**	**0.58**	**0.59**	**0.61**	**0.47**	**0.48**	**0.35**	**0.39**	0.00	0.01	0.01	***												
14	**0.73**	**0.75**	**0.71**	**0.72**	**0.74**	**0.61**	**0.62**	**0.52**	**0.56**	0.00	0.13	0.10	0.01	***											
15	**0.75**	**0.77**	**0.73**	**0.75**	**0.77**	**0.64**	**0.64**	**0.54**	**0.59**	0.05	0.19	0.15	0.06	0.00	***										
16	**0.78**	**0.79**	**0.76**	**0.78**	**0.80**	**0.66**	**0.66**	**0.57**	**0.62**	0.05	0.20	0.16	0.07	0.00	0.00	***									
17	**0.69**	**0.71**	**0.67**	**0.69**	**0.71**	**0.57**	**0.57**	**0.46**	**0.51**	0.00	0.10	0.08	0.00	0.00	0.00	0.00	***								
18	**0.77**	**0.79**	**0.75**	**0.77**	**0.79**	**0.65**	**0.65**	**0.56**	**0.60**	0.06	0.20	0.15	0.08	0.00	0.00	0.00	0.01	***							
19	**0.78**	**0.79**	**0.77**	**0.78**	**0.80**	**0.68**	**0.68**	**0.59**	**0.63**	0.06	0.22	0.18	0.08	0.00	0.00	0.00	0.00	0.00	***						
20	**0.67**	**0.69**	**0.64**	**0.66**	**0.69**	**0.53**	**0.54**	**0.42**	**0.46**	0.00	0.05	0.03	0.00	0.00	0.02	0.00	0.00	0.03	0.02	***					
21	**0.74**	**0.76**	**0.71**	**0.74**	**0.77**	**0.60**	**0.61**	**0.50**	**0.55**	0.03	0.15	0.11	0.04	0.00	0.00	0.00	0.00	0.00	0.00	0.01	***				
22	**0.66**	**0.68**	**0.63**	**0.64**	**0.66**	**0.54**	**0.54**	**0.43**	**0.46**	0.00	0.07	0.05	0.00	0.00	0.03	0.03	0.00	0.04	0.04	0.00	0.01	***			
23	**0.77**	**0.78**	**0.75**	**0.77**	**0.79**	**0.65**	**0.65**	**0.56**	**0.60**	0.05	0.19	0.15	0.07	0.00	0.01	0.00	0.00	0.00	0.00	0.01	0.00	0.03	***		
24	**0.75**	**0.77**	**0.72**	**0.75**	**0.78**	**0.61**	**0.61**	**0.51**	**0.56**	0.03	0.16	0.12	0.05	0.00	0.00	0.00	0.00	0.00	0.00	0.01	0.00	0.01	0.00	***	
25	**0.75**	**0.76**	**0.73**	**0.75**	**0.77**	**0.63**	**0.64**	**0.54**	**0.58**	0.02	0.16	0.12	0.04	0.00	0.00	0.00	0.00	0.00	0.00	0.00	0.00	0.01	0.00	0.00	***

### Genealogical reconstructions in *S. lessonii*

**Figure 3:**
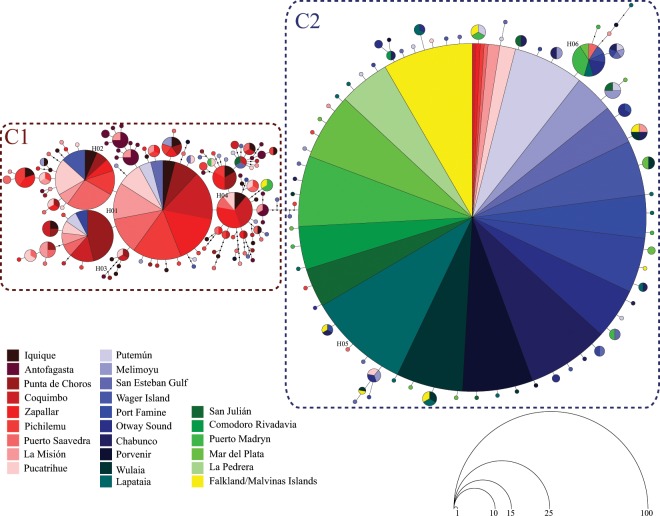
General maximum parsimony mtDNA haplotype network obtained from 625 *Siphonaria lessonii* sequences. Recorded haplotypes are represented by a colored circle indicating the locality where they were collected. The size of each circle is proportional to its frequency in the complete COI data set. For graphical purposes, main genetic clusters (C1 & C2) are confined within dashed-line rectangles.

The maximum parsimony network of mtDNA COI sequences in *S. lessonii* recorded a total of 192 haplotypes (Fig. 3) and clearly discriminated two main genetic clusters that are separated by six substitution steps (Fig. 3). The first cluster (C1) was mainly formed by individuals collected in R1 (91.1%) localities along CP, but some individuals (8.9%) from R2 (MP) also fell within C1. Similarly, the second cluster (C2) consisted of individuals from MP and AP but some individuals (9.2%) from R1 (CP) also fell within C2. Interestingly, the percentage of R1 individuals recorded in C2, as well as the percentage of R2 individuals collected in C1 decreased as we move away from the cluster’s boundary, located around 42°S (Figs. 3, S2 and S3).

As previously determined using standard diversity indices, C1 along the CP showed a more expanded genealogy than C2 across MP and AP. Several medium-frequency haplotypes (H01–H04) in C1 were broadly distributed across CP from Iquique (20°S) to Pucatrihue (40°S). Associated with these medium-frequency haplotypes we recorded many low- to single-frequency ones (n = 114). Conversely, the C2 parsimony network showed a typical star-like topology with a very short genealogy (Fig. 3). A central dominant haplotype (H05) was the most frequent in the whole data set (44%) and mainly distributed across MP and AP (Fig. 3). According to Posada and Crandall^38^, this dominant haplotype should represent the most ancestral of the Magellan diversity, whereas the most derived C2 haplotype is related to it with a maximum branch length of 5 mutational steps (Fig. 3). A medium-frequency haplotype (H06) recorded in several C2 localities was also detected in one individual from La Misión (CP). Many low- to single-frequency haplotypes surrounded the dominant Magellan haplotype (n = 72) of C2 (Fig. 3). The dominant haplotype recorded in C2 (H05) was also found in several individuals collected at R1 localities, and it was more frequent in those sites located close to the cluster boundary at 42°S (e.g. La Misión and Pucatrihue) (Fig. S2). Similarly, several individuals collected at R2 localities carried medium-frequency haplotypes from the C1 diversity (H01–H03), particularly in sites located in the MP populations located near the cluster’s boundary at 42° S (e.g. Putemún, Melimoyu, and San Esteban Gulf).

The nuclear ITS maximum parsimony network included a total of 19 different haplotypes (Fig. S3), with a similar pattern of genetic diversity and structure to those estimated for COI. The nuclear genealogy also discriminated two major genetic clusters, separated by four mutational steps (Fig. S3). Again, C1 included individuals from Iquique to Pucatrihue (PC) that exhibited a more expanded genealogy compared to those from MP and AP populations that showed a typical star-like one. The dominant C1 haplotype (H01) was widely distributed across the CP. Surrounding H01 we recorded a total of nine single-frequency haplotypes; four of them were separated by more than one mutational step (Fig. S3). A second dominant haplotype (H13) was widely distributed in C2 populations from the MP and AP, including the Malvinas/Falkland Islands. A total of six single-frequency haplotypes are closely associated with this dominant Magellan haplotype. As recorded in COI sequences, one C1 individual from la Misión in R1 (CP) shared the dominant sequence found in R2 (H13). Similarly, another individual from Melimoyu in R2 (MP) carried the dominant haplotype of C1 (H01) (Fig. S3).

### Gene flow and connectivity

**Figure 4:**
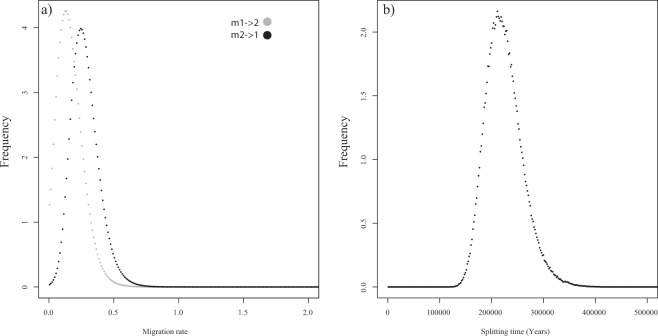
Isolation-with-migration analysis in *Siphonaria lessonii*. (**a**) Marginal posterior probability distribution of migration rates estimates between main genetic clusters C1 and C2. (**b**) Marginal posterior probability distribution of splitting time (t) estimates between main genetic clusters C1 and C2 expressed in years. Splitting time was scaled into years using a 1% per million years substitution rate modified with a ten-fold correction following Ho^39^.

Maximum likelihood gene flow analysis using MIGRATE with different migration models detected evidence of low levels of gene flow with signals of being asymmetric from the CP (C1) towards the MP + AP (C2). Among the tested models, the one from C1 to C2 received the highest probability (Table 4). Similarly, the coalescent approach of isolation-with-migration implemented in IMA2 detected overall low levels of gene flow between C1 and C2 (Table 5). Nevertheless, the model predicted a most likely pattern of asymmetric migration with high and significant migration from C1 to C2 (LRT, 10.092) (Fig. 4A, Table 5), and low and non-significant migration (LRT, 2.583) from C2 to C1 (Fig. 4A, Table 5). IMA2 analyses also estimated that the separation between C1 and C2 occurred between 100,000 and 200,000 years ago, using a mutation rate between 1 and 2% (Fig. 4B, Table 5).

**Table 4:** Thermodynamic integration (T.I.) and log Bayes factor (LBF) comparisons for different migration models between the main genetic clusters recorded in *Siphonaria lessonii* (C1 and C2).

Model	T.I.	LBF	Model prob	Model rank
1. full migration	−5274.456	−367.701	<0.0001	3
**2. C1 to C2**	−**5090.605**	**0**	**1**	**1**
3. C2 to C1	−5151.506	−121.802	<0.0001	2
4. panmixia	−5659.569	−1137.927	<0.0001	4

**Table 5:** Estimates of migration rates (m) between the recorded *Siphonaria lessonii*’s main genetic clusters in each direction, genetic diversities (Θ) of each group, ancestral diversity before splitting (Θ_A_) and splitting time of both groups escalated in years.

Value	Θ_A_	Θ1	Θ2	t0 (Years)	m_1->2_	m_2->1_
HiPt	357.5	128.5	21.5	211050.7246	0.135	0.245
Mean	399.2	133.1	26.09	224094.2029	0.1682	0.2791
HPD95Lo	218.5	89.5	8.5	156702.8986	0	0.075
HPD95Hi	607.5	180.5	47.5	298007.2464	0.355	0.505
LRT					2.583 ns	10.092***

Analyses were based on the isolation-with-migration algorithm implemented in IMa2. High Point (HiPt), mean and 95% highest posterior densities (95Lo and 95Hi) of the marginal posterior probabilities are shown. Significant patterns in asymmetrical migration rates were calculated using a Likelihood Ration Test (LTR). Significant values of m of the LRT are marked with asterisks: *P < 0.05, **P < 0.01, P < 0.001.

### Demographic inference in *S. lessonii*

**Figure 5:**
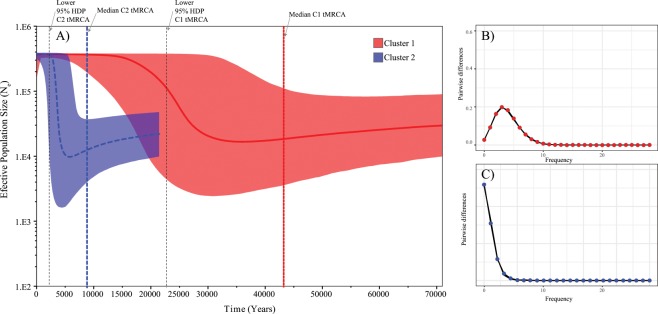
Demohraphic reconstruction in *Siphonaria lessonii*. (**A**) Bayesian Skyline plot mtDNA reconstructions of historical effective population sizes of *Siphonaria lessonii* across its distribution. The y-axis is the product of effective populations size (N_e_) and generation length in a log scale and the x-axis in the time in years. The median estimate (red solid line for the Chile-Peru Province cluster and blue dashed line for the Magellan and Argentinean province cluster) and 95% highest posterior density (HPD) limits (red for Chile-Peru province and blue for Magellanic and Argentina provinces) are shown. The thick dashed lines represent the time of the most recent ancestor (tmrca) for the Chile-Peru province cluster (red) and for the Magellanic-Argentina province cluster (blue). The thin dashed lines correspond to the lower 95% HDP of the tmrca. Distributions of pairwise differences for the mtDNA gene COI in *Siphonaria lessonii* populations from (**B**) the Chile-Peru province (C1) and (**C**) the Magellan and Argentina provinces (C2). X-axis = Pairwise differences and y-axis = frequency.

According to the Bayes factor analysis, the uncorrelated relaxed clock with exponential distribution is the model that best fitted our data with the HKY + I + G estimated by jModelTest. Bayesian skyline plot analyses resulted in different demographic trajectories for the genetic clusters recorded in *S. lessonii* across its distribution in southern South America. Populations in the Chile-Peru province showed a more stable and older demographic history than those of the Magellan and Argentinian provinces, which showed evidence of a very recent expansion (Fig. 5A). Bayesian skyline plot analyses allowed us a rough estimation for the time of the most recent common ancestors (tmrca) and the timing of population expansions between the recorded genetic groups in *S. lessonii* (Fig. 5A). Time estimates using a population-based mutational rate suggest that the tmrca for C1 may have occurred around 43 ka, while the trmca of C2 would have occurred about 8 ka. Moreover, the onset of population expansion in C1 was dated around 35 ka while in C2 it took place approximately 5 ka. As expected for star-like genealogies, global Tajima’s D and Fu’s F neutrality tests were both negative and significant for each of the recognized clusters and for the whole COI data set (Table 2). Considering the main patterns of genetic diversity and structure recorded in *S. lessonii*, the distribution of pairwise differences varies considerably between the main genetic clusters. The mismatch distribution of C1 was unimodal (Fig. 5B) while the distribution of pairwise differences in C2 showed a typical L-shaped distribution (Fig. 5C).

## Discussion

How species and ecosystems responded to Quaternary glacial cycles can tell us about the evolution and biogeography of the biota during the last million years and provide useful information for understanding their responses to future climate change^1–4,7–9,30,40–43^. In this study we recorded differences in the genetic signals of the pulmonate *S. lessonii* between temperate and high latitude South American areas, probably associated with contrasting Quaternary glacial histories among the areas analyzed, as well as with the ecology of the species (i.e. habitat preferences, reproductive mode and bathymetric range). A concordance between historic biogeographic and contemporary phylogeographic patterns supports that evolutionary forces determining species distributions are also associated with spatial patterns of populations genetic structure^5,6,18,44,45^. Here we corroborate previous molecular results in the species^24,28^, and enhance our understanding of the Quaternary biogeography of the species by recognizing two clearly discriminated genetic clusters currently found in: 1) temperate areas across the Chile-Peru province, north of the 42°S in the Pacific, and 2) cold and temperate areas across the Magellan and Argentina provinces, respectively. The boundary of these clusters (C1 and C2) agrees with the 42°S biogeographic break demonstrated in several marine organisms^36,46–50^. This break represents the southern limit for many Chile-Peru Province species and the northern border for numerous Magellan ones^13,36^. The shift in species composition recorded in marine organisms at 42°S in the southeast Pacific has been explained by changes in salinity and wave exposure^48,51^, topographical shifts of the coastline as a consequence of the increased number of fjords and channels^10,11,47^, differences in main oceanographic patterns^13,18,19,36,50^, and Quaternary glaciological histories^18,19,27,43^. In fact, this biogeographic limit coincides with the separation of the West Wind Drift into two main Pacific currents, the Humboldt Current System flowing north across the Central Chilean coast and the Cape Horn Current heading south along the Pacific Magellan margin^36,46^. Our results support that 42°S represents a main boundary in the biogeography of *S. lessonii* in southern South America, as recorded in other marine invertebrates including patellogastropods^14^, polychaetes^49^ and macroalgae^48^.

Colonization toward former glacial areas involves a series of genetic bottlenecks, and accordingly recolonized areas should exhibit low genetic diversity dominated by few haplotypes and high frequency of sequences identical to or descended from the founding population^1,4,6,38^. The hypothesis predicting that non-glaciated areas should exhibit higher levels of genetic diversity and complexity than those sites that were heavily impacted during Quaternary glacial cycles is well supported here for the southeast Pacific, where populations from the Chile-Peru Province (R1) showed higher levels of genetic diversity than those located south of the 42°S break (R2), across areas that were heavily impacted by ice during glacial maxima. Thus the main phylogeographic signatures in *S. lessonii*, together with patterns of genetic diversity, structure, connectivity, and demographic reconstructions provide clear evidence in favor or the E-C model of Quaternary biogeography^3^. Nevertheless, contrary to our expectations, populations along the Atlantic margin of the species distribution, an area considered less impacted by ice during glacial periods, did not exhibit higher genetic diversity than those located within heavily glaciated areas in the Pacific margin of the Magellan province. Populations along temperate areas of the Argentinean province also showed low levels of genetic diversity and represent a continuity of the diversity found along the Magellan province. Absence of genetic differentiation between areas that were differentially affected by ice expansion and contraction along the Pacific and Atlantic margins of Magellan was also recorded in the pulmonate *Siphonaria lateralis*^21^ and in the keystone patellogastropod *Nacella magellanica*^20,23^.

During the LGM the sea level was between 120 and 140 m below its present level^52^; a large portion of the Atlantic Continental Shelf (ACS) was exposed as a consequence of these glacio-eustatic movements, with the development of enormous plains^10^. A plausible explanation for the genetic pattern recorded in *S. lessonii* and in other hard-bottom invertebrates across the Atlantic portion of their distributions relies on the fact that during the LGM the coastline lay 200 to 400 km away and was mainly dominated by depositional environments. Accordingly, it is likely that suitable hard substrate habitats for these species would have been strongly reduced and dominated by abrasion platforms of friable sedimentary rocks^53^. During the LGM, sandy environments dominated over the entire ACS while gravels and rocks were more abundant in southern areas^54^. Gravel environments covered around 12.5% of the total ACS and were very abundant south of 46°S off the coasts of Santa Cruz and Tierra del Fuego in Argentina and in the Malvinas/Falkland Islands. Bedrock outcrops during the LGM covered 2% of the total ACS and also were more abundant in southern Atlantic areas of Magellan^53^. Accordingly, suitable refuges for *S. lessonii* would have been more available in the southern tip of the southwest Atlantic during the LGM, from where the species could have recolonized lower latitudes following the deglaciation process and the sea level increment, as proposed by Trovant *et al*.^43,44^ for the scorched mussel *Perumytilus purpuratus*. Thus current phylogeographic patterns in higher latitude near-shore marine invertebrates are not solely due to the impact of Quaternary glacial ice expansion and contraction but also to the availability of suitable habitats for the species.

Another interesting result recorded in *S. lessonii* is the absence of genetic structure along the Chile-Peru province (R1), as well as along the Magellan-Argentina provinces (R2). Such results may be a consequence of gene flow mediated by larval dispersal through the main flows of the Humboldt Current System, heading north along the Chilean coast, as well as the Cape Horn Current, heading south across the Magellan province, as recorded in several cold and temperate near-shore marine organisms^19–21,55,56^. Strong demographic and geographic expansion following post-glacial sea level rise may also be a suitable explanation for the low levels of genetic diversity and the high degree of genetic homogeneity in *S. lessonii* across these provinces. This pattern of genetic structure has been found in several Magellan marine benthic near-shore organisms with dispersive potential including invertebrates^20,55^, vertebrates^14,22^ and macroalgae^19,27^. Finally, in contrast to recent phylogeographic analyses in sub-Antarctic invertebrates including *Siphonaria* species^21^, isopods^57^, and patellogastropods^20,55^, we found in *S. lessonii* a complete absence of genetic structure between continental Magellan and the Malvinas/Falkland Islands populations. The presence of a free-living dispersive stage in *S. lessonii*, absent in other sub-Antarctic *Siphonaria* species, seems to play a key role establishing genetic homogeneity across thousands of kilometers from Chiloé Island to Uruguay in the southwest Atlantic. Nevertheless, it is important to mention that the degree of resolution of the analyzed mitochondrial and nuclear markers does not allow us to distinguish whether the low divergence across these provinces are due to contemporary connectivity or is only a consequence of post-glacial demographic expansion.

**Figure 6:**
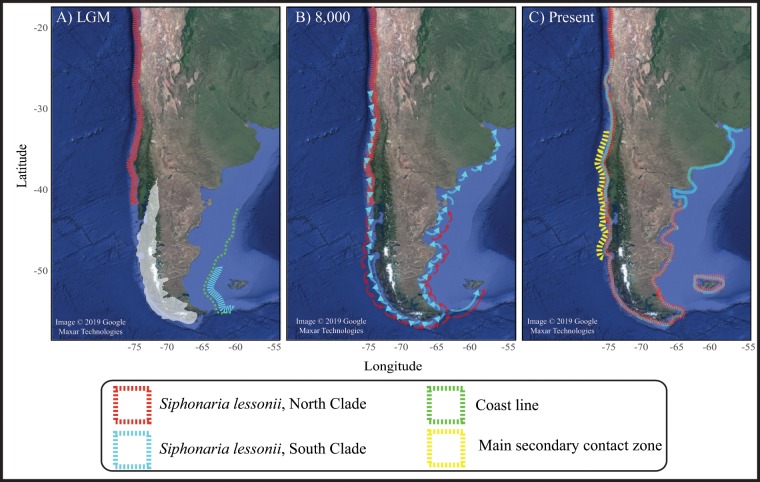
Hypothethical events leading to the current pattern of phylogeographic signal in *Siphonaria lessonii* across its distribution in southern South America. (**A**) During the Last Glacial Maximum (LGM) the northern clade was confined to warm-temperate regions of the southeastern Pacific while the southern clade was restricted to the cold-temperate Magellan province. (**B**) During the interglacial period the northern clade colonized the Magellan province while the southern clade expanded its distribution northward into the Argentina and westward to the Chile-Peru provinces, respectively. (**C**) Currently, both clades are broadly distribution in southern South America but a main secondary contact zone (SCZ) is recognized between the two clades. Map from Google earth Pro V 7.3.2.5776 (March 5, 2019). South America. −37.9313°S; −69.7831°W, Eye alt. 4210.9 Km. SIO, NOAA. Image Landset Copernicus. Data LDEO-Columbia, NSF, NOOA. http://www.earth.google.com [Dec 13, 2015].

Overall, our demographic time estimates (trmca and population expansions) should be tempered by the consideration of potential effects of three different sources of uncertainty including (a) evolutionary variability around the coalescence; (b) inadequate samplings, which may have missed some haplotypes and led to underestimates of population ages and (c) uncertainty of the molecular clock of the mtDNA COI^16^. Of these three, we recognize evolutionary variability around the coalescence as the main important source of uncertainty. Incomplete sampling of haplotypes is inevitable in any study of natural populations but the extensive geographical coverage we obtained for *S. lessonii* should ensure that most of the common haplotypes were included. Unsampled haplotypes are probably rare and terminal ones and should not likely affect the overall shape of the skyline plot or the isolation-with migration analyses. However, they may have some effect on the time of coalescence. Finally, the substitutional rate used in demographic inference analyses will also greatly determine the time estimation of population growth detected here. Nonetheless, even considering important confidence interval around the substitution rate used here, our estimations allow us to infer that divergence onset between clades and demographic events in *S. lessonii* were associated to Quaternary glacial processes and probably to the last glacial cycle. The paleogeographic evolution of the Magellan continental shelves (Pacific and Atlantic), together with genealogical and demographic reconstructions, as well as main patterns of genetic diversity/structure and gene flow analyses in *S. lessonii* allow us to propose a Quaternary biogeographic hypothesis for the species across its distribution in southern South America. At the end of the last interglacial period, the geographic distribution of *S. lessonii* was similar to that currently observed and extended from northern Chile to Uruguay. During glacial maxima, ice expansion over the Pacific Magellan coastline would have eradicated the species from this area, while populations in non-glaciated Pacific areas north of the 42°S break would have persisted along the Chile-Peru province. Along the Atlantic margin, a reduction of suitable habitats during glacial maxima would have led to local extinction of species associated with these hard bottom habitats, as recorded in other invertebrates during the Holocene such as the keystone Magellan gastropod *Tegula atra*^58,59^. As proposed by Aguirre *et al*.^59^ the absence of this important Magellan hard-bottom species along the Argentine Atlantic represents a climate-change driven shift associated with changes in Sea Surface Temperature (ca. 2 °C higher), wind velocities (less), light (less), nutrient availability (less), and intensity of cold (less) and warm (increased) shallow water currents that altered water masses and biogeographical boundaries in the region. Paleo-eustatic reconstructions in the southwest Atlantic Patagonia suggest that during glacial maxima a higher proportion of suitable habitats for *S. lessonii* at higher latitudes in the Atlantic Magellanic margin^53,54^ may have allowed the persistence of small populations that acted as glacial refugia located close the southern tip of South America (Fig. 6A). At the end of the last glacial period, ice sheet contraction along Pacific Magellan shore and sea-level rise along the Atlantic margin would have uncovered new suitable habitats, allowing a stepwise recolonization towards lower latitudes (Fig. 6B) until *S. lessonii* recovered its present distribution (Fig. 6C). Separated since the onset of the last glacial period, populations isolated in the Atlantic refugia diverged from the genetic cluster recorded in the Chile-Peru province. Evidence of the separation of these clusters is the presence of at least six mutation steps between them, suggesting that they remained apart for a significant period of time. Following this, the southern clade (C2) re-expanded from the southern Atlantic margin towards the Pacific and to lower latitudes in the Atlantic following the deglaciation process around 8,000 years ago (Fig. 6B). Similarly, the northern clade (C1) expanded its distribution southward across the Magellan province to Puerto Madryn and the Malvinas/Falkland Islands in the Atlantic. Currently, the two main genetic clusters (C1 and C2) recorded in *S. lessonii* are broadly distributed across the species distribution but they are found in higher frequency between 34°S and 46°S, producing a secondary contact zone with introgression when the clusters came back into contact (Figs. 6C and S2).

Phylogeographical studies in South American near-shore marine benthic organisms are helping us to understand the recent evolution of higher latitude marine biota. Main phylogeographic signatures in *S. lessonii* along the Pacific margin of its distribution provide obvious evidence towards the E-C model of Quaternary biogeography proposed by Provan & Bennett^3^. In contrast, temperate area populations across the Argentina and Atlantic Patagonia showed very low levels of genetic diversity and structure and are clearly connected to the diversity recorded across the Pacific Magellan margin. Accordingly, the impact of Quaternary ice expansions over the demography of near-shore species does not solely explain current patterns of genetic diversity across the Atlantic Magellan margin and the Argentinean Province. Populations of *S. lessonii* across these areas that were less impacted by ice during glacial maxima share the diversity and structure that probably survived the last glacial maxima at higher latitudes refugia. Accordingly, ecological factors such as the availability of suitable habitats, bathymetry, developmental mode and the likelihood of dispersal through rafting are also key factors explaining main phylogeographical patterns in the species across its distribution in different South American ecoregions.

## Methods

### Sampling, DNA extraction, PCR amplification and sequencing

Individuals were sampled from 25 localities across the distribution of *S. lessoni* in the Chile-Peru Province (CP), the Magellan Province (MP), and the Argentina Province (AP) (Fig. 1; Table 1).

Specimens were preserved in ethanol (99%) and DNA was prepared from the mantle using standard salting-out methodology following Aljanabi & Martinez^60^. Universal primers *LCO1490* and *HCO2198*^61^ and *its-1d* and *its-4r*^62^ were used to amplify partial fragments of the mitochondrial gene cytochrome c oxidase subunit I (COI) and the nuclear internal transcribed spacer region (ITS1 and ITS2), respectively. PCR procedures were performed using 2.5 μl 10X Buffer (Invitrogen), 1.0 μl of 50 mM MgCl_2_ (Invitrogen), 200 mM dNTPs (Invitrogen), 10 pg/μl each of dATP, dGTP, dCTP and dTTP (Invitrogen), 1 U Taq polymerase (Invitrogen), 50 ng of genomic DNA and 16.7 μl of double-distilled water. Thermal cycling parameters for the analyzed markers followed González-Wevar *et al*.^21^. PCR products were purified using QIAquick Gel Extraction Kit (QIAGEN) and sequenced in both directions with an ABI3730 x 1 Automatic Sequencer at Macrogen Inc. (Seoul, South Korea).

### Genetic diversity and population structure in *S. lessonii*

Mitochondrial (COI) and nuclear (ITS) data sets of *S. lessonii* were edited independently using GENEIOUS^63^ and aligned with MUSCLE^64^. New COI and ITS sequences have been deposited in GenBank under the following accession numbers: MN436071–MN436695 (COI), and MN436696–MN436714 (ITS). In order to evaluate how saturation of transitions is accumulated in relation to divergence we performed a saturation analysis in DAMBE 5^65^. Following Wright^66^, we evaluated the codon usage of mtDNA sequences using the effective number of codon value (ENC) in DNASP v.5^67^.

Polymorphism of mtDNA sequences was estimated in DNASP using standard diversity indices: number of haplotypes (*k*), segregating sites (*S*), haplotype diversity (*H*), average number of pairwise differences (*П*), and nucleotide diversity (*π*) per site, genetic cluster, and for the whole COI data set.

Levels of genetic structure between populations of *S. lessonii* were estimated following Pons and Petit^68^ using main pairwise differences (N_ST_) and haplotype frequencies (G_ST_) in ARLEQUIN v. 3.5^69^. The statistical significance of pairwise population comparisons was determined through permutation tests (20,000 iterations) of haplotype identities following Excoffier *et al*.^69^. We also determined levels of phylogeographic differentiation using the nearest-neighbor statistic (S_nn_) in DNASP and the statistical significance of the analyses was determined using a permutation test (10,000 iterations) following Hudson^70^.

The spatial mtDNA genetic structure of *S. lessonii* across its distribution was inferred using two clustering methods. First, through a Bayesian model we determined the number and composition of panmictic groups and the spatial boundaries among them using GENELAND v. 2.0.0^71^ in the R environment (R, version 2.4.1)^72^. The best association of localities was estimated using a Markov Chain Monte Carlo (MCMC) procedure including genetic and geographic information following Guillot *et al*.^71^. Analyses were run using 50 × 10^6^ MCMC iterations sampled each 1,000 steps. Stationarity of the analyses was identified in TRACER v.1.5 (http://beast.bio.ed.ac.uk/Tracer) and 10% of the iterations were removed as burn-in. A maximum number of clusters (K = 24) was included in the analyses to determine model parameters and posterior probabilities of group membership. Second, using SAMOVA we determined the composition and number of geographically homogeneous and maximally differentiated groups of localities following Dupanloup *et al*.^73^. This analysis allows characterization of spatial genetic structure by separating the variance into within populations, among populations within groups and among groups. This methodology seeks to maximize the proportion of total genetic variance due to differences among groups.

### Genealogical reconstructions, gene flow and connectivity in *S. lessonii*

Genealogical relationships in *S. lessonii* were constructed based on mtDNA (COI) and nucDNA (ITS) sequences using maximum parsimony networks in HAPVIEW^74^. Different models of gene flow between the mtDNA groups were tested for different scenarios using the software MIGRATE v.3.5 following Beerli & Felsenstein^75^. Four candidate models were compared constraining the presence of directionality of gene flow between the genetic groups found in *S. lessonii*. A first model permitted bidirectional gene flow (full-island model). Models 2 and 3 were defined based on the asymmetry of gene flow between groups. Finally, the fourth model assumed the groups were a single panmictic population. The analyses were performed using a TrN + I + G substitution model and transition-transversion ratio of 16.9927, estimated using JMODELTEST v2^76^. The specific substitution rate for the selected marker in siphonariids^77,78^, was set to constant, following Darriba *et al*.^76^. The analysis included a long chain with 500,000 recorded parameter steps with a sampling interval of 100 and a burn-in of 10%. Following Darriba *et al*.^76^ we used a heated scheme of 1.0, 1.5, 3.0. and 1000000.0 to estimate the marginal likelihoods of the models to compared them. A thermodynamic integration approximation (T.I.) was used for the log-equivalent Bayes Factor (LBF) analyses following Beerli & Palczewski^79^. The associated probability of each model in relation to another was estimated through Bayes Factors following Kass and Rafery^80^. We also determined different demographic indices to evaluate asymmetric isolation-with-migration models between the recognized genetic groups using IMA2 software^39,81^. For this we performed several preliminary runs in the M-mode (Markov Chain Monte Carlo; MCMC mode) to estimate the best set of priors. Uniform priors were used to determine the effective population size (Θ_1_, Θ_2_, and ancestral Θ_a_, Θ = 600) and splitting time (t = 30); an exponential prior (mean = 20) for gene flow (m) was assumed. We performed 80 × 10^6^ MCMC steps sampling every 100 generations, with a burn-in period of 10%. Mitochondrial sequences in *S. lessonii* were assumed to mutate under the HKY mutation model following Hey and Nielsen^81^. Simulated genealogies were performed under the L-Mode (Load Tree mode) to determine the log maximum likelihood and credibility interval (95% under HPD) estimates for migration parameters using a likelihood ratio test. Finally, under the L-Mode mtDNA data set of *S. lessonii* was compared against a null model of no migration^39,81^.

### Demographic inference in *S. lessonii*

Demographic mtDNA dynamics were determined using a Bayesian skyline plot (BSP) methodology implemented in the program BEAST v.1.8^82^. For this we performed three independent Bayesian Monte Carlo analyses for 100 × 10^6^ generations. Trees and parameters were sampled every 1,000 generations in each of the genetic groups of *S. lessonii*. Previously, JMODELTEST was used to estimate the proper substitution model using the Akaike information criterion (AIC). Following Ho *et al*.^83^ we used a rough tenfold evolutionary rate correction for population-based analyses. A time-dependency rate of molecular evolution has been described where the short-term mutation rate (1 to 2 Ma) could be tenfold higher than the long-term substitution rate used for phylogenetic reconstructions. Accordingly, BSP analyses used a 10% population-based mutation rate considering the 1.0% phylogenetic rate used for siphonariids^77,78^. Convergence between runs was confirmed with TRACER and the results for multiple runs were combined using LOGCOMBINER v1.4.7^84^.

We performed statistical neutrality tests (Tajima’s D and Fu’s F_S_) using DNASP for the whole COI data set and for each recognized genetic group to estimate whether sequences deviate from mutation-drift equilibrium. Demographic reconstructions were also estimated using the comparison of pairwise differences between haplotypes (mismatch distribution) for each recognized group to the expected distribution under the sudden expansion growth model^85^. For this we estimated three main parameters: i) τ = date of population expansion/contraction measured in units of mutational time (τ = 2 μt) where t = time in years and μ = mutational rate per sequence per year, ii) initial theta *θ*_*i*_ = *2 N*_*i*_*μ* before the population expansion/contraction), and iii) final theta *θ*_*t*_ = *2 N*_*t*_*μ* after population expansion/contraction. Finally, demographic parameters were calculated in DNASP using a nonlinear least square approach following Schneider & Excoffier^86^.

## Supplementary information

Supplementary Information.

## Data Availability

New COI and ITS sequences in *Siphonaria lessonii* will be available in GenBank under Accession Numbers MN436071–MN436695 (COI), and MN436696–MN436714 (ITS1 & ITS2).
