# Supplementary material for: Quaternary ice sheets and sea level regression drove divergence in a marine gastropod along Eastern and Western coasts of South America
Source: Sci Rep. 2020 Jan 21;10:844. doi: 10.1038/s41598-020-57543-4 (PMC6972712; doi:10.1038/s41598-020-57543-4)

***Scientific Reports***

**SUPPORTING INFORMATION**

**Quaternary ice sheets and sea level regression drove divergence in a marine gastropod along Eastern and Western coasts of South America**

**AUTHORS:** Fernández Iriarte PJ, González-Wevar CA, Segovia NI, Rosenfeld S, Hüne M, Fainburg L, Nuñez JD, Haye PA, Poulin E.

**Figure S1.** General maximum parsimony mtDNA haplotype network obtained from 625 *Siphonaria lessonii* sequences comparing cluster membership (C1 & C2) vs region of collection (R1 & R2). Where R1 = Chile-Peru province and R1 = Magellanic + Argentina provinces.


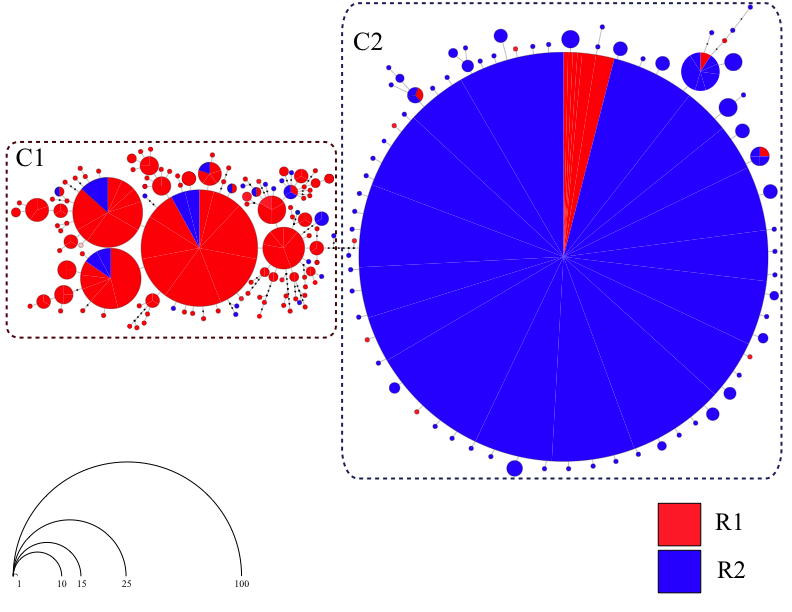


**Figure S2.** Proportion of *Siphonaria lessonii* specimens belonging to genetic cluster C1 (red) and genetic cluster C2 (blue) in each locality across the species distribution in South America.


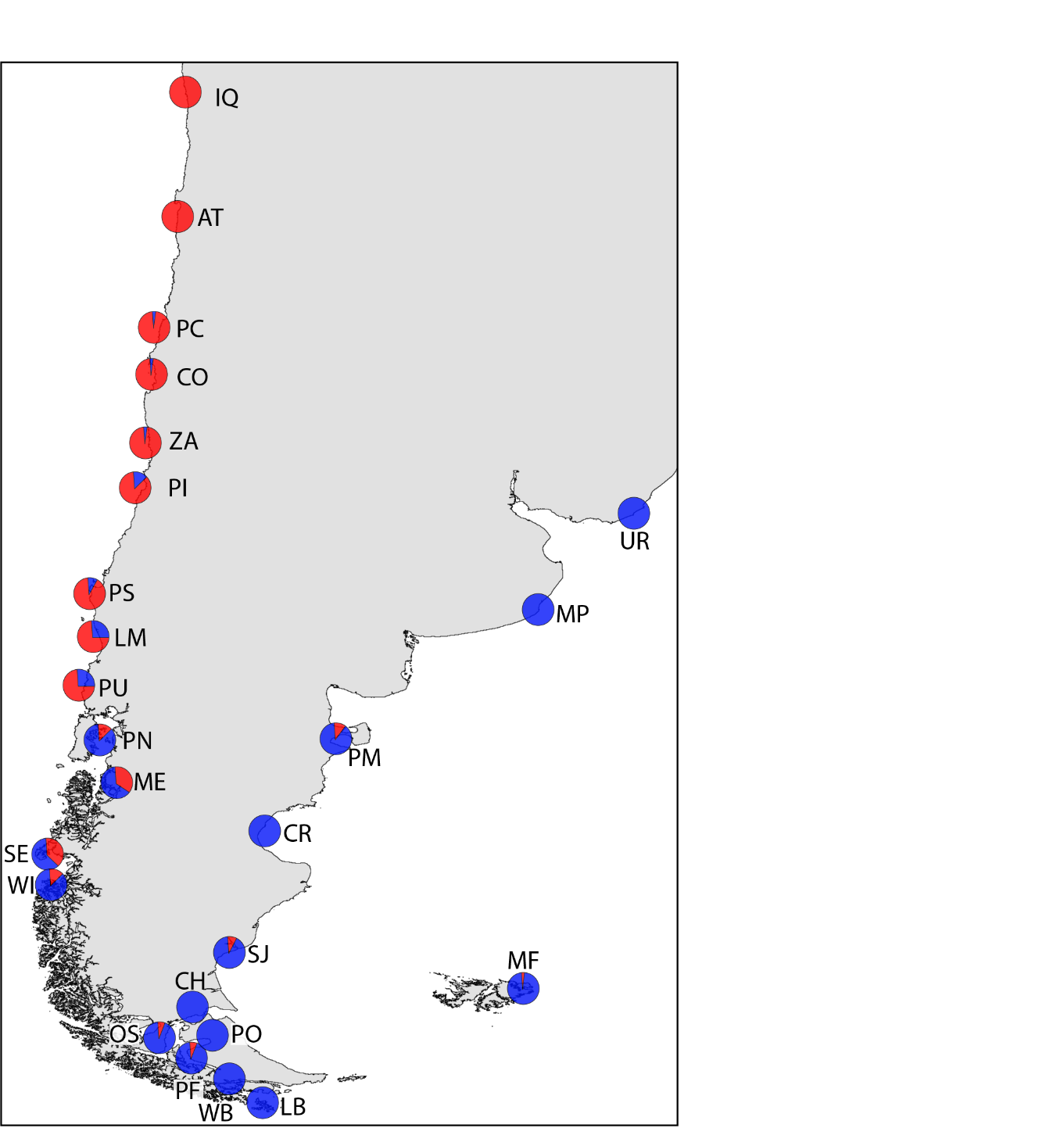


**Figure S3.** General maximum parsimony nucDNA (ITS1 & ITS2) network obtained from 45 *Siphonaria lessonii* individuals. Sequences are represented by a colored circle indicating the locality where they were collected. The size of each circle is proportional to its frequency in the complete ITS data set. For graphical purposes, the main genetic clusters (C1 & C2) are confined within dashed-line rectangles.


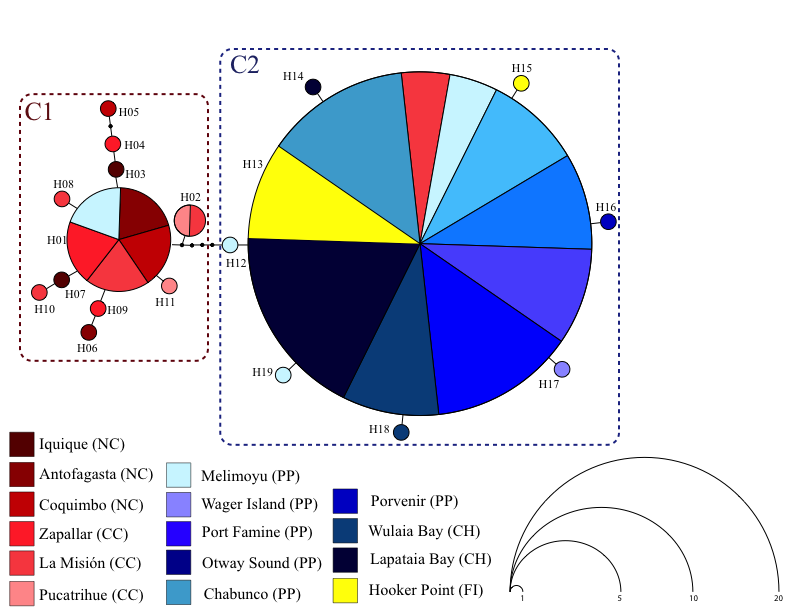

Supplement: Supplementary file 1 — Supplementary Information. [file 41598_2020_57543_MOESM1_ESM.docx]
